# Paranoid Thinking as a Function of Minority Group Status and Intersectionality: An International Examination of the Role of Negative Beliefs

**DOI:** 10.1093/schbul/sbad027

**Published:** 2023-03-21

**Authors:** J L Kingston, B Schlier, T Lincoln, S H So, B A Gaudiano, E M J Morris, P Phiri, L Ellett

**Affiliations:** Department of Psychology, Royal Holloway, University of London, Bowyer, UK; University of Hamburg, Institute for Psychology, Department of Clinical Psychology and Psychotherapy, Von-Melle-Park 5, 20146, Hamburg, Germany; University of Hamburg, Institute for Psychology, Department of Clinical Psychology and Psychotherapy, Von-Melle-Park 5, 20146, Hamburg, Germany; Department of Psychology, The Chinese University of Hong Kong, Hong Kong SAR, China; Department of Psychiatry & Human Behavior, Brown University and Butler Hospital, Providence, RI, USA; School of Psychology & Public Health, La Trobe University, Bundoora, Melbourne, Australia; Southern Health NHS Foundation Trust, Botley Rd, West End, UK; School of Psychology, University of Southampton, Southampton, UK; School of Psychology, University of Southampton, Southampton, UK

**Keywords:** paranoia, minority group, negative beliefs, positive beliefs, social rank

## Abstract

**Background:**

Paranoia is higher in minority group individuals, especially those reporting intersecting aspects of difference. High negative and low positive self and other beliefs, and low social rank, are predictive of paranoia overtime; however, data are typically from majority group participants. This study examined whether social defeat or healthy cultural mistrust best characterizes paranoia in minority groups.

**Study Design:**

Using cross-sectional, survey design, with a large (*n* = 2510) international sample, moderation analyses (PROCESS) examined whether self and other beliefs, and perceived social rank, operate similarly or differently in minority vs majority group participants. Specifically, we tested whether beliefs moderated the influence of minority group, and intersecting aspects of difference, on paranoia.

**Study Results:**

Paranoia was consistently higher in participants from minority vs majority groups and level of paranoid thinking was significantly higher at each level of the intersectionality index. Negative self/other beliefs were associated with elevated paranoia in all participants. However, in support of the notion of healthy cultural mistrust, low social rank, and low positive self/other beliefs were significantly associated with paranoia in majority group participants but *unrelated* to paranoia in respective minority group members.

**Conclusions:**

Although mixed, our findings signal the need to consider healthy cultural mistrust when examining paranoia in minority groups and bring into question whether “paranoia” accurately describes the experiences of marginalized individuals, at least at low levels of severity. Further research on paranoia in minority groups is crucial to developing culturally appropriate ways of understanding people’s experiences in the context of victimization, discrimination, and difference.

## Introduction

Social context plays an important role in understanding psychotic experiences in clinical and general population samples,^1,2^ with social adversity (eg, migration, bullying, discrimination) significantly increasing the risk of psychosis^3^ and psychotic experiences.^1,4–6^ However, disparate social adversities are often grouped together, which may obscure specific associations and nuances. Likewise, focusing on broad diagnostic categories (eg, schizophrenia) can lack sensitivity to the heterogeneous nature of individual symptoms (eg, paranoia, hallucinations). Perhaps most importantly, the extent to which theoretical ideas derived from majority group individuals (eg, predominantly white, able bodied, heterosexual) are valid and relevant to minority group individuals is seldom examined. Current cognitive and social defeat models^7,8^ suggest that subordination, exclusion, and powerlessness enhance paranoia via heightened negative and lower positive self and other beliefs, as well as low perceived social rank. However, the healthy cultural mistrust hypothesis suggests that “paranoia” in minoritised groups may reflect a healthy, adaptive response to increased exploitation and discrimination.^9^ To explore this empirically, this study examines the association between singular and co-occurring (intersecting) minority group status(es) and paranoid beliefs in an international general population sample. Specifically, we test whether high negative and low positive beliefs about the self and others and low perceived social rank have common or different associations with paranoia across 5 minority groups and their intersection.

Paranoia describes exaggerated and unfounded beliefs that others intend to cause you harm.^10^ Paranoia is thought to exist on a continuum, ranging from mild suspicion and mistrust that is likely to have some adaptive value, to persecutory delusions, which describe more extreme, distressing, and persistent paranoid beliefs.^11^ Paranoia is common in the general population with weekly prevalence rates of up to 40%, and approximately 20% reporting high levels of conviction and distress.^12^ Paranoid delusions (ie, plots to harm you) are less common, with estimated lifetime prevalence of 0.7%, rising to 36% in those reporting a lifetime psychotic experience.^13^ Social context is important for understanding paranoia. Paranoia is inherently interpersonal,^14^ with theoretical models^8,15,16^ suggesting that adverse experiences (eg, victimization, trauma) give rise to unduly generalized beliefs about the risk posed to oneself by others, especially when individuals report negative self and other beliefs.^17^ Paranoia may be more influenced by adverse social contexts than other psychotic-like experiences. For example, social deprivation (eg, unemployment, barriers to housing) is associated with paranoia, but not hallucinations or hypomania^18^; perceived ethnic discrimination is more associated with paranoia than other psychotic symptoms^19^; and discrimination across a variety of minority groups prospectively predicted delusional ideation, but not hallucinations, over a 3-year period in the general population.^20^

The social defeat hypothesis proposes that feeling subordinate, like an outsider, or excluded from the majority group is a social context associated with enhanced risk for psychotic experiences, including paranoia.^8,21^ For example, in a UK general population sample, perceived discrimination due to minority group status was associated with increased rates of psychotic experiences (eg, paranoia, hypomania, auditory hallucinations).^22^ Importantly, paranoia was significantly elevated across diverse minority groups, suggesting that the risk may not be specific to any one group. In individuals from racially minoritised groups, heightened risk of psychotic experiences can be ameliorated by living in an area where there is a greater proportion of individuals from your own ethnic group.^23^ This, along with similar findings^24^ suggests that the extent to which you feel different from others in your social environment is important for understanding increased vulnerability to psychotic experiences. The specific nature of difference (ie, sexuality, race) may be less central. Intersectionality,^25^ grounded in the agenda for social justice, describes the way in which co-occurring marginalized identities can compound discrimination and oppression. Although conceived to promote social action, intersectionality has important implications for mental health, with research showing that intersecting aspects of difference are associated with accumulating risk for mental health difficulties, including paranoia.^26^ For example, perceived discrimination across multiple marginalized identities was associated with increased odds for experiencing paranoia (OR = 6.00) relative to discrimination in a singular domain (OR = 2.27),^22^ which echoes findings on delusional thinking^20^ and on the cumulative effect of multiple social adversities.^27^

A core assumption of cognitive models^7^ is that self and other beliefs play an important role in understanding the impact of social adversity on paranoia. For example, Jaya et al.^1^ found that the link between social adversity (including, but not restricted to, discrimination, and minority group status) and psychotic symptoms in the general population was mediated by high negative and low positive beliefs about self and others and low perceived social rank. However, because minority group status was grouped with other social adversities, possible differences across minority groups were not examined. Most of what we know about the cognitive model of psychotic experiences, and the central role of cognition in psychotic experiences, is based on majority group samples. As of yet, an unexplored question is whether these negative beliefs about self and others operate equivalently across minority and majority groups. Cultural relativity,^9^ for example, suggests that different cultural groups experience symptoms in fundamentally different ways, underscoring the need to understand experiences within their cultural context. In a clinical high-risk group, Rouhakhtar et al.^28^ examined whether race (Black vs White participants) moderated the effect of psychotic experiences on social functioning and found that in White participants, psychotic experiences predicted social functioning, whilst in Black participants the two were unrelated. Item-level analysis suggested that paranoia and hallucinations drove this moderation effect. Investigating cultural mistrust in treatment-seeking African Americans, Whaley^29^ also raises the issue that cultural mistrust towards White clinicians may contribute to diagnostic errors, whereby mistrust arising from cultural difference is misinterpreted as clinical paranoia. Together, these findings support the view that, at the general population level, “paranoia” in ethnic minority groups may reflect adaptive responding or “healthy cultural mistrust”^8,30^ rather than being a manifestation of problematic belief systems.

The main aim of this study was to internationally investigate the link between minority group status and paranoia, across a variety of minority groups, examining whether minority status and cognitive factors interact in their association with paranoia. Based on aforementioned research and theory, we hypothesized that:

1. Paranoia will be significantly higher in minority vs majority group participants across five formally dissimilar minority groups and across five culturally dissimilar sites.
2. Paranoia scores will systematically increase with the number of intersecting minority identities an individual reports.
3. Finally, we tested whether self/other beliefs and perceived social rank moderate the impact of minority/majority group status on paranoia. Social defeat and cognitive models suggest that high negative and low positive self/other beliefs and low social rank *moderate the strength* of the association between majority/minority group status and paranoia. Alternatively, the healthy cultural mistrust hypothesis implies that self/other beliefs and perceived social rank may be unrelated to paranoia in minority groups. Given the lack of research in this area, we did not favor one hypothesis over the other.

## Methods

### Design

We used a cross-sectional survey design with minority group status/intersectionality index as the hypothesized predictor, paranoid beliefs as the outcome, and positive and negative beliefs about the self/others and perceived social rank as hypothesized moderators. Co-variates included age, gender, education, general distress, and site.

### Participants

The sample consisted of 2510 participants from United Kingdom (*n* = 512), United States (*n* = 502), Germany (*n* = 516), Hong Kong (*n* = 445), and Australia (*n* = 535). Participants were recruited using Qualtrics panel recruitment using stratified quota sampling at each site based on sex, age, and educational attainment. The sample was therefore drawn from a self-selecting group (ie, registered to take part in Qualtrics studies), with imposed stratified quotas to ensure that the age, gender, and education level were representative of the population at each site.

### Power Considerations

This research constitutes a re-analysis of an existing dataset, so no a priori power calculations concerning the minority/majority group analyses were performed before collecting the original data. Post hoc computation of achieved power showed that for comparisons between majority and minority groups with a 90%/10% distribution of group status, the test power was *β* = 0.85 for small effects (*d* = 0.2) and approximately *β* ≈ 1.00 for medium effects (*d* = 0.5).

## Measures

This article is based on a larger survey^31^ and, as such, additional measures were taken but are not here reported.

*Sociodemographic variables.* Participants provided information on their age, gender, and education.

*Minority group status.* ^1^ Participants viewed a list of 5 minority groups and ticked where applicable: minority sexual orientation/identity, one or more physical disabilities, belonging to an ethnic minority or having a different skin color to the majority of people living around you, having a minority religion/belief, and having a visible physical condition (eg, obese, mole, scar). We used the item-wise responses for analyzing the effects of individual minority group status effects and summed positive answers to create an *intersectionality index* (range: 0 = “member of none of the aforementioned minorities”; 5 = “member of all 5 of the aforementioned minorities”).

*The Revised Green et al. Paranoid Thoughts Scale—Persecution (R-GPTS* ^32^) uses 10 items to measure paranoid thinking over the last 2 weeks. Items are rated on a 0 (not at all) to 4 (totally) scale (range 0–40), and higher scores indicate higher levels of paranoid thinking. Scores of ≤5 are average, 6+ is elevated, 11+ is moderately severe, 18+ is severe and 28+ is very severe (α = 0.96).

*The Brief Core Schema Scales (BCSS* ^33^) is a 24-item 5-point (0 to 4) self-report measure of evaluative self and other beliefs. The BCSS yields 4 sub-scores: negative self, positive self, negative others, and positive others (α’s all >0.85).

*The Social Comparison Scale (SCS* ^34^ *),* used to measure social rank, consists of 11 bi-polar items (eg, inferior-superior, outsider-insider) which participants rate from 1 (eg, inferior) to 10 (eg, superior) using the response stem “in relation to others I feel….” Items are rated over the last 4 weeks and higher scores thus indicate a more positive view of oneself in relation to others (α = 0.95).

*The short version of the Depression Anxiety Stress Scales (DASS* ^35^) are a 21-item self-report measure of the clinically significant negative emotional states of depression, anxiety, and tension/stress over 1 week using a scale from 0 (did not apply to me at all) to 3 (applied to me very much, or most of the time). For this study, we used the global sum-score as an indicator of general distress (α = 0.96).

### Procedure

Ethical approval was obtained from all university host sites. Qualtrics sent an email invitation to *n* =12 853 potential participants, of which *n* = 2510 fulfilled quota and eligibility checks, consented and completed the survey, and passed attention, and time checks (see Lincoln et al. for further details).^36^ Questionnaires were completed online and participants were reimbursed for taking part.

### Analysis Strategy

We used *t*-tests to test the first hypothesis. Additionally, linear regression models were calculated to test for the robustness of differences when controlling for general distress (DASS-21) and sociodemographic variables (site, age, gender, and level of education). To test hypothesis 2, we calculated a random intercept multilevel-regression model (participants nested in sites) with the intersectionality index as a continuous, independent variable and paranoia as the dependent variable. Again, robustness of the results was tested by entering sociodemographic variables (minus site) and distress as control variables. To test hypothesis 3, we computed 6 random intercept multilevel-regression models for each of the minority groups, as well as for the intersectionality index, entering one of the putative moderators and testing for the group status × moderator interaction effect, respectively. For each of the 30 multilevel-regression models, a Generalized Information Matrix Test (GIM)^37^ for site-clustered standard errors was calculated to test for substantial deviation from homoscedasticity. If GIM tests were significant, robust standard errors were used for the respective model (see supplementary materials for details).

## Results

### Sample Characteristics

Average age was 43.3 years (SD = 15.7). Most participants described themselves as female (52.5%), 46.9% as male, 0.2% as genderqueer, 0.1% as TransMale/TransFemale, and 0.2% as “other.” For highest education achievement: most had bachelor’s degree/equivalent (31.3%), 30.8% A-level/equivalent, 24.5% secondary school, 10% master’s degree/equivalent, 1.5% PhD/equivalent, 1.9% primary school. About half the sample worked full time (50.9%), 14.2% worked part time, and 11.3% were unemployed. The remaining participants were retired (7.9%), in job training or school (6.7%), home keeper/carer (5.7%), or disabled (3.0%). Annual income: 19.3% under £18 500, 28.6% £18 500–£36 999, 20.8% £37 000–£55 999 12.3% £56 000–£74 999 and 18.0% > £75 000 per year. Lifetime diagnosis of a mental health disorder was reported by 21%, with 16.2% currently taking medication for this. Minority group status ranged from 9.44% (ethnic minority) to 18.53% (visible physical condition; table 1).

**Table 1.** Comparison of Mean Paranoid Thinking in Different Minority Groups (vs Corresponding Majority Group)

		Majority Group			Minority Group			*T*-Test	
Minority Group type	Sample	*N*	*M*_Paranoia_	SD_Paranoia_	*N*	*M*_Paranoia_	SD_Paranoia_	*T*	*d*
Ethnicity	All	2273	7.22	9.91	237	13.14	11.96	8.56***	0.58
	UK	452	5.57	9.37	60	9.55	11.14	2.65**	0.41
	US	481	6.11	9.81	54	13.44	13.16	3.97***	0.72
	Australia	445	11.31	10.83	57	17.53	10.96	4.04***	0.57
	Germany	487	5.26	8.69	29	13.45	12.14	3.58**	0.92
	Hong Kong	408	8.25	9.5	37	11.49	11.1	1.72^+^	0.34
Religion	All	2255	7.23	9.92	255	12.67	11.84	8.14***	0.54
	UK	468	5.76	9.49	44	8.95	11.05	1.86^+^	0.33
	US	470	6.22	9.99	65	11.38	12.25	3.25**	0.50
	Australia	443	11.18	10.68	59	18.25	11.55	4.46***	0.66
	Germany	470	5.52	8.97	46	7.8	10.27	1.46	0.25
	Hong Kong	404	7.75	9.29	41	16.15	10.20	5.07***	0.90
Sexual identity/orientation	All	2243	7.15	9.80	267	13.07	12.31	9.06***	0.59
	UK	451	5.42	9.13	53	10.56	12.12	3.95***	0.54
	US	482	5.75	9.46	53	16.87	13.16	5.99***	1.12
	Australia	447	11.38	10.67	55	17.11	12.49	3.26**	0.53
	Germany	463	5.20	8.65	53	10.32	11.53	3.14**	0.57
	Hong Kong	400	8.32	9.63	45	10.31	10.00	1.27	0.21
Physical disability	All	2202	7.40	9.90	308	10.51	12.20	5.00***	0.30
	UK	466	5.67	9.22	46	9.72	12.96	2.07*	0.42
	US	455	6.31	9.92	80	9.94	12.54	2.46*	0.35
	Australia	420	12.31	10.80	82	10.49	12.00	-1.28	-0.17
	Germany	455	5.2	8.51	61	9.61	12.09	2.76**	0.49
	Hong Kong	406	7.99	9.37	39	14.05	11.07	3.31**	0.64
Visible physical condition	All	2045	7.15	9.92	465	10.56	11.25	6.52***	0.33
	UK	445	5.96	9.74	67	6.49	9.21	0.43	0.05
	US	440	6.45	10.17	95	8.68	11.38	1.76^+^	0.21
	Australia	419	11.69	10.76	83	13.63	12.15	1.35	0.18
	Germany	402	4.63	8.11	114	9.58	11.19	4.41***	0.56
	Hong Kong	339	6.97	8.88	106	13.46	10.46	5.77***	0.70

*Note*: ***P < .001 **P < .01 *P < .05 ^+^*P* < .1.

### Paranoid Thinking in Different Minority Groups

**Fig. 1.**
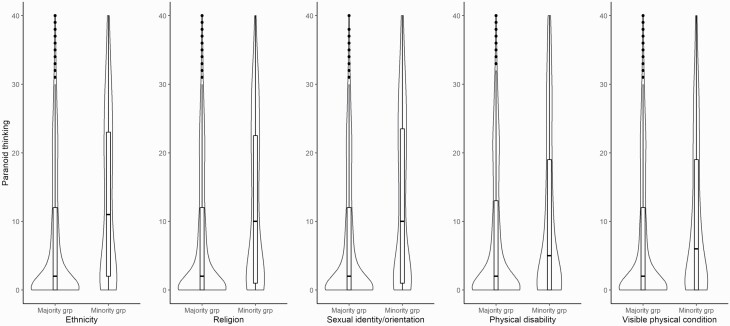
Boxplots and Violinplots of the distribution of paranoid thinking in minority and corresponding majority groups.

In the full sample, all *t*-tests comparing minority to corresponding majority groups showed significant differences, with all minority groups showing higher levels of paranoid thinking compared to the majority (table 1). For minority groups based on ethnicity, religious beliefs, or sexual identity/orientation, these differences amounted to moderate effects (0.54≤*d*≤0.59), whereas the effects were smaller for physical disability and visible physical condition (0.30≤*d*≤0.34). As can be seen in the violin plots in figure 1, the distribution of paranoia scores in the majority group was highly skewed, with most participants reporting low levels of paranoia (ie, scores 0–10). In the minority groups, the distributions of paranoia scores were overall less skewed, with a tendency towards a bi-modal distribution for ethnicity, religion, and sexual identity/orientation.

When controlling for general distress and demographic variables using multiple regression, minority vs majority group differences remained significant for ethnicity (*b* = 2.36, SE = 0.55, *t* = 4.33, *P* < .001) religious beliefs (*b* = 1.89, SE = 0.53, *t* = 3.59, *P* < .001), and sexual identity/orientation (*b* = 1.18, SE = 0.53, *t* = 2.25, *P* = .025). For physical disability (*b* = 0.22, SE = 0.49, *t* = 0.44, *P* = .658) and visible physical conditions (*b* = 0.69, SE = 0.41, *t* = 1.68, *P* = .094), however, the effects became nonsignificant when control variables were entered into the regression models. Furthermore, as can be seen in table 1, testing for difference within each site yielded comparable results with all but one comparisons showing higher paranoia scores in the minority group. Whereas 7 of the 25 site-specific *t*-tests did not reach significance, nonsignificant results did not cluster at any one site.

### Paranoid Thinking as a Function of Intersecting Minority Group Status

**Fig. 2.**
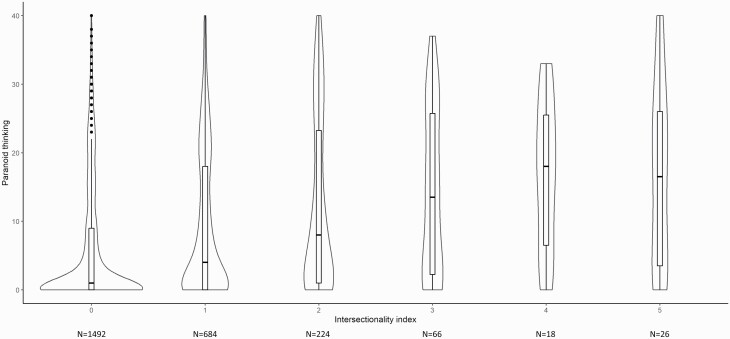
Boxplots and Violinplots of the distribution of paranoid thinking at all levels of the intersectionality index.

Next, to examine whether paranoia is systematically higher with increasing endorsement of minority group membership, the intersectionality index was entered as a continuous predictor of paranoid thinking in a random intercept multilevel-regression model, showing a significant effect (*b* = 2.71, SE = 0.21, *t* = 13.03, *P* < .001). As can be seen in figure 2, the distribution of paranoid thinking scores changed from a skewed distribution with a low mean score at low index levels to a progressively more normal distribution up until index level 4. Furthermore, entering the intersectionality index as a factor showed that each level of the intersectionality index had a significantly higher level of paranoid thinking (index level 1: *b*_1_ = 3.14, SE = 0.45, *t* = 7.03, *P* < .001; index level 2: *b*_2_ = 6.21, SE = 0.70, *t* = 8.93, *P* < .001; index level 3: *b*_3_ = 8.43, SE = 1.22, *t* = 6.92, *P* < .001; index level 4: *b*_4_ = 10.78, SE = 2.30, *t* = 4.70, *P* < .001; index level 5: *b*_5_ = 9.93, SE = 1.92, t = 5.18, *P* < .001). Finally, the intersectionality index effect remained significant when controlling for demographics and general distress (*b* = 0.75, SE = 0.18, *t* = 4.23, *P* < .001).

### Effect of Putative Self/Other Beliefs and Perceived Social Rank in Minority vs Majority Groups

Finally, we tested for moderation (table 2). None of the GIM tests for any of the models was significant, so no robust standard errors were used (supplementary materials). All main effects of moderator variables were significant showing that in the majority group, higher levels of negative beliefs, lower levels of positive beliefs, and lower perceived social rank were all associated with higher levels of paranoid thinking. For the interaction effects (ie, different effects for minority group participants), a more complicated picture emerged.

**Table 2.** Overview of Main and Interaction Effects for Moderation Analyses Based on Random Intercept Multilevel Regression (Participants Nested in Sites)

	Sexual Identity/Orientation		Ethnicity		Religion		Physical Disability		Visible Physical Condition		Intersectionality Index	
Moderator	Main Effect^a^	Inter-action^b^	Main Effect^a^	Inter-action^b^	Main Effect^a^	Inter-action^b^	Main Effect^a^	Inter-action^b^	Main Effect^a^	Inter-action^b^	Main Effect^a^	Inter-action^b^
Neg. beliefs (self)	0.75***	−0.17	0.76***	−0.13	0.72***	0.08	0.74***	0.04	0.71***	0.09	0.68***	0.01
Neg. beliefs (other)	0.65***	0.04	0.65***	0.00	0.65***	−0.01	0.63***	0.17*	0.63***	0.11	0.58***	0.05
Pos. beliefs (self)	−0.18***	0.22*	−0.19***	0.27**	−0.17***	0.10	−0.16***	0.08	−0.13***	0.00	−0.17***	0.07*
Pos. beliefs (other)	−0.29***	0.28**	−0.29***	0.20	−0.28***	0.19	−0.27***	−0.03	−0.25***	−0.04	−0.27***	0.06*
Social rank	−0.07***	0.10***	−0.07***	0.07*	−0.06***	0.02	−0.067***	−0.01	−0.05***	−0.01	−0.06***	0.01

*Note*:

^a^The main effect constitutes the effect of the respective moderator variable (eg, social rank) in the majority group,

^b^The interaction effect shows the difference from the main effect (majority) in the minority group. Significance levels: ***P < .001; **P < .01; *P < .05.

First, for *negative beliefs about oneself and others,* almost no significant interaction effects were found. This suggests that the significant main effect found for the majority group generalizes to the respective minority groups. The only exception was negative beliefs about others × minority status due to physical disability (*b* = 0.17, SE = 0.08, *t* = 2.21, *P* = .027), which showed that for people with a physical disability, the effect of negative beliefs about others on paranoid thinking was more pronounced than for individuals without a physical disability.

**Fig. 3.**
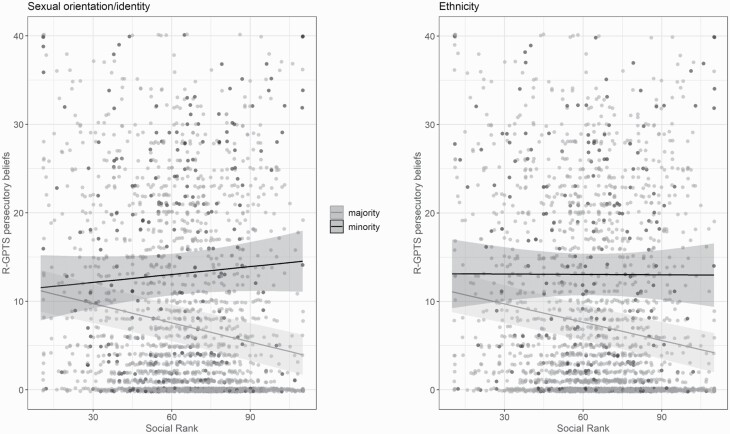
Exemplary depiction of the association between social rank and paranoid thinking as a function of ethnic (left) and sexual identity/orientation (right) group status.

For *social rank* and for *positive beliefs about the self and others*, we found significant interaction effects with ethnicity (social rank: *b* = 0.07, SE = 0.03, *t* = 2.15, *P* = .031, beliefs about self: *b* = 0.27, SE = 0.10, *t* = 2.72, *P* = .006) and sexual identity/orientation (social rank: *b* = 0.11, SE = 0.03, *t* = 3.61, *P* < .001; beliefs about self: *b* = 0.21, SE = 0.09, *t* = 2.39, *P* = .011; beliefs about others: *b* = 0.28, SE = 0.10, *t* = 2.98, *P* = .003). All these interaction effects, however, were in the *opposite* direction compared to the main effect, indicating *no effect* of social rank or positive beliefs about the self and others (figure 3) in the respective minority group.

**Fig. 4.**
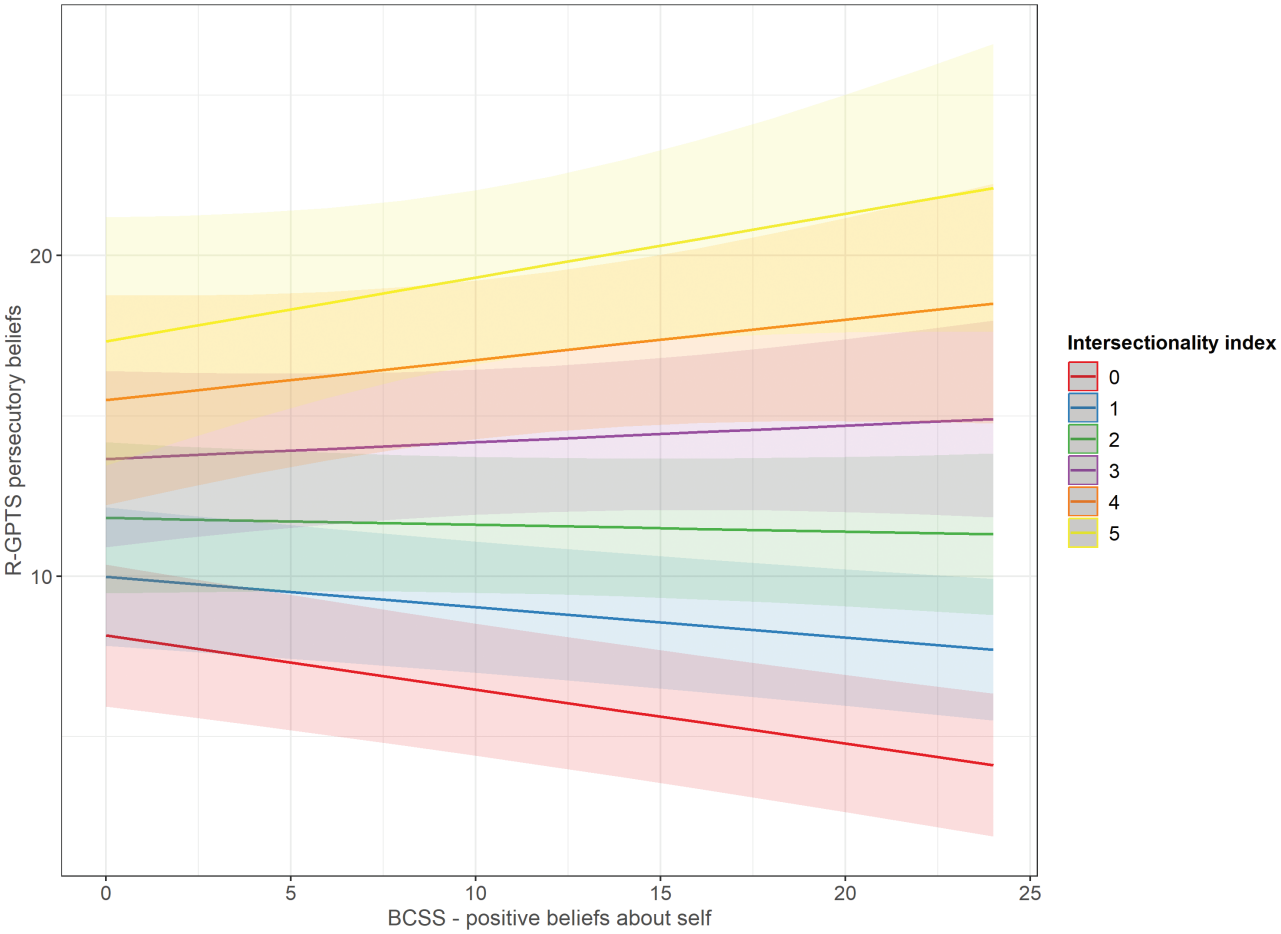
Predicted associations between positive beliefs and paranoid thinking as a function increasing intersectionality index levels. Linear regression by intersectionality index level with 95% confidence bands.

There was also a significant interaction for the effect of *positive beliefs about the self and others*, on the intersectionality index (self: *b* = 0.07, SE = 0.03, *t* = 2.30, *P* = .021; others: *b* = .06, SE = 0.03, *t* = 2.03, *P* = .043). Figure 4 provides a visual example for the change in the association of positive beliefs (about the self) and paranoia with increasing intersectionality index levels: at low levels (0–1), the association tends to be negative. At medium levels (eg, 3) there seems to be no association, whereas highest levels of the intersectionality show a positive association between positive beliefs and paranoia by descriptive values. However, confidence bands at these highest levels are larger (due to small subsamples at high levels of the intersectionality index) and include *b* = 0, indicating no significant positive association.

## Discussion

Consistent with previous research,^6^ paranoia scores were consistently higher in participants from minority vs majority groups. When controlling for distress, site, and sociodemographic factors, this remained the case for minority sexual, ethnic, and religious groups only. Intersecting aspects of difference had cumulative effects on paranoia; each level of the intersectionality index had a significantly higher level of paranoid thinking, which remained significant when controlling for potential confounds. Findings are consistent with previous research showing elevated paranoia in participants experiencing minority group discrimination and the cumulative impact of intersectionality on paranoia/delusional thinking.^20,22^ Our findings show for the first time that these associations hold in an international sample, across geographically and culturally diverse sites. Taken together, and consistent with previous research,^23,24^ these findings suggest that being different from others in your social environment is associated with higher paranoia scores. Furthermore, whereas previous research has focused more on the delusional end of the paranoia continuum, our data show similar patterns of association at lower-severity levels. This adds to a substantial literature supporting common predictors and causal mechanisms across the continuum of experience.^38^

A key aim of this study was to use moderation analysis to test whether social defeat or healthy cultural mistrust best characterizes paranoia in minority groups. Consistent with cognitive models,^7^ we found that for individuals who did not identify as belonging to the respective minority group, high negative and low positive self and other beliefs and low perceived social rank were all associated with higher levels of paranoia. This confers with existing research identifying that these cognitive factors enhance paranoid thinking.^1^

Interestingly, however, interactions showed a more nuanced set of findings. Firstly, for *negative* beliefs about self/others, all but one interaction (physical disability) was nonsignificant, indicating that negative beliefs about self/others had an almost ubiquitous effect across participants, irrespective of majority/minority group status or site. This finding coheres with the existing evidence base to stress the importance of extreme negative self and other beliefs in understanding paranoia and in its assessment and treatment. Counter to the social defeat view, there was no evidence that this effect was greater for minority vs majority group participants except for the case of physical disability, where the effect of negative other beliefs on paranoid thinking was more pronounced in those with, vs without, a physical disability. Secondly, for physical disability, visible physical condition, and religious minority, there were no interactions for positive beliefs or social rank either. These results suggest that for these groups, cognitive factors had a common role across majority/minority groups.

Perhaps the most interesting and important findings arose from interactions in ethnic and sexual identity/orientation minority groups. In all instances, low social rank and low positive self/other beliefs were associated with higher paranoia scores in majority group participants but were *unrelated* to paranoia in respective minority group members. This suggests that for participants from sexual and ethnic minority groups, paranoia operated independently from positive beliefs about self/others and ones perceived rank in society. The corresponding pattern occurred for positive self/other beliefs and the intersectionality index: With increasing levels of the intersectionality index, there was a gradual shift from the majority-conform association between positive beliefs and paranoia towards no association. These findings are consistent with Rouhakhtar et al.^28^ who reported that paranoia was associated with social functioning in White, but not Black, participants. Whilst our findings are mixed (ie, convergence for negative beliefs, yet divergence for positive beliefs and social rank), and require replication, they nonetheless suggest that “paranoia” in ethnic and sexual minority groups may require a fundamentally different interpretation from paranoia in respective majority groups. Our data could suggest that paranoia in ethnic and sexual minority groups may reflect healthy and adaptive threat beliefs, arising from lived experiences of victimization, discrimination, and social inequality.^29^ Ethnic and sexual minority individuals have long been recognized as facing significant health disparities in terms of risk factors, access to healthcare, and health outcomes, which is compounded in those with minoritised identities plus psychotic experiences.^39,40^ Our findings underscore the importance of formulation driven conceptualisations of paranoia that incorporate minority group status and intersectionality to help understand an individual’s experience of paranoia in context.^41^

This study has several limitations. Minority status was assessed by a simple yes/no question. We therefore cannot provide any further information regarding specific minority groupings (eg, whether the effects are similar for Asian vs Black vs White minorities), and the use of self-report offers no external validation of reported status. The data are also cross-sectional and temporal association between variables cannot be determined. This is a priority area for future research. For social rank, participants were asked to consider themselves relative to others in general. In doing so, it is not possible to ascertain whether participants brought to mind those in their in or out group, which will have been key to the responses given. Obtaining a clearer account of this in future research would be helpful. Finally, our exclusive focus on high-income countries is an important limitation. Future research on low-/middle-income countries is needed.

In sum, in this diverse international sample, paranoia was elevated across all minority groups, suggesting this may be ubiquitous at least to high-income countries. Intersecting aspects of difference were associated with systematically higher levels of paranoia, suggesting a cumulative effect. Consistent with social defeat and cognitive models, negative self/other beliefs were associated with higher paranoia scores irrespective of majority/minority group status. However, low positive self/other beliefs and low social rank were associated with paranoia in majority group participants but were unrelated to paranoia in sexual and ethnic minority participants. Findings provide partial support for the healthy cultural mistrust hypothesis in participants from ethnic and sexual minority groups and could suggest the need to reconceptualize “paranoia” in these marginalized groups. For now, our findings highlight the importance of considering minority group status when making sense of people’s interpersonal threat beliefs and caution against making assumptions based on research from predominantly majority group participants.

## Supplementary Material

sbad027_suppl_Supplementary_Material

## References

[1] Jaya ES , Ascone L, Lincoln TM. Social adversity and psychosis: the mediating role of cognitive vulnerability. Schizophr Bull. 2017;43:557–565. 27451429 10.1093/schbul/sbw104 PMC5463978

[2] Van Os J , Kenis G, Rutten BP. The environment and Schizophrenia. Nature. 2010;468:203–212. 21068828 10.1038/nature09563

[3] Kirkbride JB , Errazuriz A, Croudace TJ, et al. Incidence of schizophrenia and other psychoses in England, 1950–2009: a systematic review and meta-analyses. PLoS One. 2012;7:e31660. doi:10.1371/journal.pone.0031660. 22457710 10.1371/journal.pone.0031660 PMC3310436

[4] Bentall RP , de Sousa P, Varese F, et al. From adversity to psychosis: pathways and mechanisms from specific adversities to specific symptoms. Soc Psychiatry Psychiatr Epidemiol. 2014;49(7):1011–1022. 24919446 10.1007/s00127-014-0914-0

[5] Linscott RJ , Van Os J. An updated and conservative systematic review and meta-analysis of epidemiological evidence on psychotic experiences in children and adults: on the pathway from proneness to persistence to dimensional expression across mental disorders. Psychol Med. 2013;43(6):1133–1149. 22850401 10.1017/S0033291712001626

[6] Pearce J , Rafiq S, Simpson, J, et al. Perceived discrimination and psychosis: a systematic review of the literature. Soc Psychiatry Psychiatr Epidemiol. 2019;54:1023–1044. 31236631 10.1007/s00127-019-01729-3

[7] Garety PA , Kuipers E, Fowler D, Freeman D, Bebbington PE. A cognitive model of the positive symptoms of psychosis. Psychol Med. 2001;31(2):189–195. 11232907 10.1017/s0033291701003312

[8] Selten JP , Van Der Ven E, Rutten BP, Cantor-Graae E. The social defeat hypothesis of schizophrenia: an update. Schizophr Bull. 2013;39(6):1180–1186. 24062592 10.1093/schbul/sbt134 PMC3796093

[9] Whaley AL. Cultural mistrust and the clinical diagnosis of paranoid schizophrenia in African American patients. J Psychopathol Behav Assess. 2001;23(2):93–100.

[10] Freeman D , Garety P. Advances in understanding and treating persecutory delusions: a review. Soc Psychiatry Psychiatr Epidemiol. 2014;49(8):1179–1189. 25005465 10.1007/s00127-014-0928-7 PMC4108844

[11] Elahi A , Algorta GP, Varese F, McIntyre JC, Bentall RP. Do paranoid delusions exist on a continuum with subclinical paranoia? A multi-method taxometric study. Schizophr Res. 2017;190:77–81. 28318838 10.1016/j.schres.2017.03.022

[12] Freeman D , Garety, PA, Bebbington PE, et al. Psychological investigation of the structure of paranoia in a non-clinical population. Br J Psychiatry. 2005;186(5):427–435. 15863749 10.1192/bjp.186.5.427

[13] McGrath JJ , Saha S, Al-Hamzawi A, et al. Psychotic experiences in the general population: a cross-national analysis based on 31 261 respondents from 18 countries. JAMA Psychiatry. 2015;72(7):697–705. 26018466 10.1001/jamapsychiatry.2015.0575 PMC5120396

[14] Ellett L , Foxall A, Wildschut T, Chadwick P. Dispositional forgiveness buffers paranoia following interpersonal transgression. J Pers. 2022. 10.1111/jopy.12755 35837856

[15] Bentall RP , Corcoran R, Howard R, Blackwood N, Kinderman P. Persecutory delusions: a review and theoretical integration. Clin Psychol Rev. 2001;21(8):1143–1192. 11702511 10.1016/s0272-7358(01)00106-4

[16] Freeman D , Garety PA, Kuipers E, Fowler D, Bebbington PE. A cognitive model of persecutory delusions. Br J Clin Psychol. 2002;41(4):331–347. 12437789 10.1348/014466502760387461

[17] Humphrey C , Bucci S, Varese F, Degnan A, Berry K. Paranoia and negative schema about the self and others: a systematic review and meta-analysis. Clin Psychol Rev. 2001;90:102081. 10.1016/j.cpr.2021.102081 34564019

[18] Wickham S , Taylor P, Shevlin M, Bentall RP. The impact of social deprivation on paranoia, hallucinations, mania and depression: the role of discrimination social support, stress and trust. PLoS One. 2014;9(8):e105140. 25162703 10.1371/journal.pone.0105140 PMC4146475

[19] Bardol O , Grot S, Oh H, et al. Perceived ethnic discrimination as a risk factor for psychotic symptoms: a systematic review and meta-analysis. Psychol Med. 2020;50(7):1077–1089. 32317042 10.1017/S003329172000094X

[20] Janssen I , Hanssen M, Bak ML. Discrimination and delusional ideation. Br J Psychiatry. 2003;182(1):71–76. 12509322 10.1192/bjp.182.1.71

[21] Selten JP , Cantor-Graae E. Social defeat: risk factor for schizophrenia? Br J Psychiatry. 2005;187(2):101–102. 16055818 10.1192/bjp.187.2.101

[22] Stickley A , Oh H, Sumiyoshi T, et al. Perceived discrimination and psychotic experiences in the English general population. Eur Psychiatry. 2019;62:50–57. 31527013 10.1016/j.eurpsy.2019.08.004

[23] Veling W , Susser E, van Os J, Mackenbach JP, Selten J-P, Hoek HW. Ethnic density of neighborhoods and incidence of psychotic disorders among immigrants. Am J Psychiatry. 2008;165(1):66–73. 18086750 10.1176/appi.ajp.2007.07030423

[24] Zammit S , Lewis G, Rasbash J, Dalman C, Gustafsson JE, Allebeck P. Individuals, schools, and neighborhood: a multilevel longitudinal study of variation in incidence of psychotic disorders. Arch Gen Psychiatry. 2010;67(9):914–922. 20819985 10.1001/archgenpsychiatry.2010.101

[25] Crenshaw K. Demarginalizing the intersection of race and sex: a Black feminist critique of antidiscrimination doctrine, feminist theory and antiracist politics. Univ Chic Leg Forum. 1989;140:139–167.

[26] Jackson SD , Mohr JJ, Sarno EL, Kindahl AM, Jones IL. Intersectional experiences, stigma-related stress, and psychological health among Black LGBQ individuals. J Consult Clin Psychol. 2020;88(5):416–428. 32091225 10.1037/ccp0000489

[27] Morgan C , Kirkbride J, Hutchinson G, et al. Cumulative social disadvantage, ethnicity and first-episode psychosis: a case-control study. Psychol Med. 2008;38(12):1701–1715. 19000327 10.1017/S0033291708004534

[28] Rouhakhtar PR , Roemer C, Reeves G, Schiffman J. The associations between attenuated psychosis symptoms and functioning in Black and White youth at clinical high-risk for psychosis. Schizophr Res. 2021. 10.1016/j.schres.2021.11.032 34922800

[29] Whaley AL. Cultural mistrust of white mental health clinicians among African Americans with severe mental illness. Am J Orthopsychiatry. 2001;71(2):252–256. 11347366 10.1037/0002-9432.71.2.252

[30] Jun HJ , Nam B, Fedina L, et al. Paranoid beliefs and realistic expectations of victimization: data from the survey of police-public encounters. Schizophr Res. 2018;199:326–332. 29525461 10.1016/j.schres.2018.02.046

[31] Kingston JL , Schlier B, Ellett L, et al. The pandemic paranoia scale (PPS): Factor structure and measurement invariance across languages. Psychol Med. 2021:1–10. doi:10.1017/S0033291721004633. 10.1017/S0033291721004633 PMC8712962 34879896

[32] Freeman D , Loe BS, Kingdon D, et al. The revised Green *et al*., Paranoid Thoughts Scale (R-GPTS): psychometric properties, severity ranges, and clinical cut-offs. Psychol Med. 2001;51(2):244–253. 10.1017/S0033291719003155 PMC7893506 31744588

[33] Fowler D , Freeman D, Smith BEN, et al. The Brief Core Schema Scales (BCSS): psychometric properties and associations with paranoia and grandiosity in non-clinical and psychosis samples. Psychol Med. 2006;36(6):749–759. 16563204 10.1017/S0033291706007355

[34] Allan S , Gilbert P. A social comparison scale: psychometric properties and relationship to psychopathology. Pers Indiv Diff. 1995;19(3):293–299.

[35] Henry JD , Crawford JR. The short‐form version of the Depression Anxiety Stress Scales (DASS‐21): construct validity and normative data in a large non‐clinical sample. Br J Clin Psychol. 2005;44(2):227–239. 16004657 10.1348/014466505X29657

[36] Lincoln TM , Schlier B, Strakeljahn F, et al. Taking a machine learning approach to optimize prediction of vaccine hesitancy in high income countries. Sci Rep. 2022;12(1):1–12. 35136120 10.1038/s41598-022-05915-3 PMC8827083

[37] King G , Roberts ME. How robust standard errors expose methodological problems they do not fix, and what to do about it. Polit Anal. 2015;23(2):159–179.

[38] Bebbington PE , McBride O, Steel C, et al. The structure of paranoia in the general population. Br J Psychiatry. 2013;202(6):419–427. 23661767 10.1192/bjp.bp.112.119032

[39] Lund EM , Burgess CM. Sexual and gender minority health care disparities: Barriers to care and strategies to bridge the gap. Prim Care. 2001;48(2):179–189. 10.1016/j.pop.2021.02.007 33985698

[40] Maura J , Weisman de Mamani A. Mental health disparities, treatment engagement, and attrition among racial/ethnic minorities with severe mental illness: a review. J Clin Psychol Med Settings. 2017;24(3):187–210. 28900779 10.1007/s10880-017-9510-2

[41] Rathod S , Phiri P, Naeem F. An evidence-based framework to culturally adapt cognitive behaviour therapy. Cogn Behav Ther. 2019;12:e10.

