# Supplementary material for: Paranoid Thinking as a Function of Minority Group Status and Intersectionality: An International Examination of the Role of Negative Beliefs
Source: Schizophr Bull. 2023 Mar 21;49(4):1078–87. doi: 10.1093/schbul/sbad027 (PMC10318883; doi:10.1093/schbul/sbad027)
Supplement: sbad027_suppl_Supplementary_Material [file sbad027_suppl_supplementary_material.docx]

Table S1

Comparison of main analysis (multilevel regression), Generalized linear models (GLM) with and without robust standard errors and Generalized Information Matrix Test (GIM) results for all models involving negative beliefs about oneself

| Minority type | Predictor | Multilevel model results | | | | GLM results | | | | GLM with robust SE | | | GIM | | |
| --- | --- | --- | --- | --- | --- | --- | --- | --- | --- | --- | --- | --- | --- | --- | --- |
|  |  | b | SE | T | p | b | SE | T | p | Robust SE | Z | p | RoT | GIM test | p |
| Sexual identity/orientation | Minority status | **5.15** | **.60** | **8.56** | **<.001** | **5.08** | **.61** | **8.32** | **<.001** | **1.36** | **3.74** | **<.001** | 4.25 | 3.8*10^-5^ | ≈ 1.00 |
|  | Neg. beliefs (self) | **.75** | **.04** | **19.65** | **<.001** | **.80** | **.04** | **2.92** | **<.001** | **.14** | **5.84** | **<.001** |  |  |  |
|  | Interaction | -.17 | .11 | -1.58 | .115 | -.17 | .11 | -1.60 | .110 | .14 | -1.22 | .222 |  |  |  |
| Ethnicity | Minority status | **5.14** | **.63** | **8.15** | **<.001** | **5.27** | **.63** | **8.26** | **<.001** | **1.22** | **4.33** | **<.001** | 4.06 | 6.2*10^-5^ | ≈ 1.00 |
|  | Neg. beliefs (sel ) | **.76** | **.04** | **19.74** | **<.001** | **.81** | **.04** | **21.03** | **<.001** | **.14** | **5.72** | **<.001** |  |  |  |
|  | Interaction | -.13 | .11 | -1.22 | .221 | -.14 | .11 | -1.31 | .191 | .14 | -1.03 | .304 |  |  |  |
| Religion | Minority status | **3.85** | **.62** | **6.17** | **<.001** | **3.93** | **.63** | **6.21** | **<.001** | **.92** | **4.28** | **<.001** | 3.65 | 4.3*10^-5^ | ≈ 1.00 |
|  | Neg. beliefs (self) | **.72** | **.04** | **18.24** | **<.001** | **.77** | **.04** | **19.48** | **<.001** | **.14** | **5.34** | **<.001** |  |  |  |
|  | Interaction | .08 | .10 | .80 | .424 | .08 | .10 | .73 | .468 | .10 | .70 | .483 |  |  |  |
| Physical disability | Minority status | .82 | .60 | 1.38 | .169 | .91 | .61 | 1.50 | .134 | 1.22 | .74 | .457 | 4.80 | 4.4*10^-5^ | ≈ 1.00 |
|  | Neg. beliefs (self) | **.74** | **.04** | **18.32** | **<.001** | **.78** | **.04** | **19.30** | **<.001** | **.11** | **6.97** | **<.001** |  |  |  |
|  | Interaction | .04 | .09 | .41 | .679 | .06 | .10 | .59 | .555 | .08 | .74 | .457 |  |  |  |
| Visible physical difference | Minority status | **1.57** | **.50** | **3.17** | **.002** | **1.44** | **.50** | **2.88** | **.004** | .90 | 1.60 | .110 | 4.09 | 4.3*10^-5^ | ≈ 1.00 |
|  | Neg. beliefs (self) | **.71** | **.04** | **16.44** | **<.001** | **.76** | **.04** | **17.73** | **<.001** | **.16** | **4.93** | **<.001** |  |  |  |
|  | Interaction | .09 | .08 | 1.14 | .255 | .07 | .08 | .87 | .383 | .12 | .60 | .546 |  |  |  |
| Intersectionality index | Intersect. index | **1.92** | **.20** | **9.49** | **<.001** | **1.93** | **.21** | **9.39** | **<.001** | **.18** | **1.58** | **<.001** | 3.76 | 4.1*10^-5^ | ≈ 1.00 |
|  | Neg. beliefs (self) | **.68** | **.05** | **14.45** | **<.001** | **.73** | **.05** | **15.50** | **<.001** | **.16** | **4.46** | **<.001** |  |  |  |
|  | Interaction | .01 | .03 | .17 | .869 | .00 | .03 | .05 | .959 | .05 | .04 | .969 |  |  |  |

Note. All GIM tests were calculated with the robustSE package in R using 100 new datasets and 100 bootstraps, respectively. RoT = Rule of thumb test for necessity for GIM test (GIM test necessary if RoT>1.5). Significant results are printed in bold.

Table S2

Comparison of main analysis (multilevel regression), Generalized linear models (GLM) with and without robust standard errors and Generalized Information Matrix Test (GIM) results for all models involving negative beliefs about others

| Minority type | Predictor | Multilevel model results | | | | | GLM results | | | | | GLM with robust SE | | | | GIM | | | |
| --- | --- | --- | --- | --- | --- | --- | --- | --- | --- | --- | --- | --- | --- | --- | --- | --- | --- | --- | --- |
|  |  | b | SE | T | p | b | | SE | T | p | Robust SE | | Z | p | RoT | | GIM test | p |  |
| Sexual identity/orientation | Minority status | **5.02** | **.59** | **8.52** | **<.001** | **5.04** | | **.61** | **8.31** | **<.001** | **1.34** | | **3.76** | **<.001** | 5.89 | | 5.5*10^-5^ | ≈ 1.00 |  |
|  | Neg. beliefs (other) | **.65** | **.03** | **2.90** | **<.001** | **.64** | | **.03** | **2.64** | **<.001** | **.06** | | **1.93** | **<.001** |  | |  |  |  |
|  | Interaction | .04 | .09 | .49 | .622 | .03 | | .09 | .35 | .725 | .14 | | .22 | .825 |  | |  |  |  |
| Ethnicity | Minority status | **4.63** | **.63** | **7.35** | **<.001** | **4.81** | | **.65** | **7.45** | **<.001** | **1.25** | | **3.85** | **<.001** | 5.67 | | 6.0*10^-5^ | ≈ 1.00 |  |
|  | Neg. beliefs (other) | **.65** | **.03** | **2.99** | **<.001** | **.65** | | **.04** | **2.68** | **<.001** | **.05** | | **12.38** | **<.001** |  | |  |  |  |
|  | Interaction | .00 | .09 | -.02 | .981 | .00 | | .09 | .00 | ≈ 1.00 | .09 | | <.01 | ≈ 1.00 |  | |  |  |  |
| Religion | Minority status | **4.05** | **.62** | **6.59** | **<.001** | **4.16** | | **9,63** | **6.57** | **<.001** | **1.07** | | **3.88** | **<.001** | 5.08 | | 4.5*10^-5^ | ≈ 1.00 |  |
|  | Neg. beliefs (other) | **.65** | **.03** | **2.85** | **<.001** | **.64** | | **.03** | **2.53** | **<.001** | **.05** | | **11.93** | **<.001** |  | |  |  |  |
|  | Interaction | -.01 | .09 | -.14 | .888 | -.00 | | .09 | -.02 | .983 | .04 | | -.05 | .956 |  | |  |  |  |
| Physical disability | Minority status | **1.06** | **.58** | **1.85** | **.064** | **1.17** | | **.59** | **1.99** | **.047** | **1.39** | | **.84** | **.399** | 6.11 | | 4.8*10^-5^ | ≈ 1.00 |  |
|  | Neg. beliefs (other) | **.63** | **.03** | **19.44** | **<.001** | **.62** | | **.03** | **18,99** | **<.001** | **.07** | | **9.98** | **<.001** |  | |  |  |  |
|  | Interaction | **.17** | **.08** | **2.21** | **.027** | **.19** | | **.08** | **2.43** | **.015** | **.07** | | **2.85** | **.004** |  | |  |  |  |
| Visible physical difference | Minority status | **1.99** | **.48** | **4.15** | **<.001** | **2.03** | | **.49** | **4.14** | **<.001** | **.94** | | **2.15** | **.031** | 5.34 | | 4.3*10^-5^ | ≈ 1.00 |  |
|  | Neg. beliefs (other) | **.63** | **.03** | **18.85** | **<.001** | **.63** | | **.03** | **18.68** | **<.001** | **.08** | | **7.94** | **<.001** |  | |  |  |  |
|  | Interaction | .11 | .07 | 1.55 | .122 | .10 | | .07 | 1.41 | .158 | .10 | | 9.98 | .932 |  | |  |  |  |
| Intersectionality index | Intersect. index | **1.95** | **.20** | **9.93** | **<.001** | **2.01** | | **.20** | **9.96** | **<.001** | **.21** | | **9.71** | **<.001** | 5.24 | | 8.5*10^-5^ | ≈ 1.00 |  |
|  | Neg. beliefs (other) | **.58** | **.04** | **15.94** | **<.001** | **.57** | | **.04** | **15.65** | **<.001** | **.08** | | **7.16** | **<.001** |  | |  |  |  |
|  | Interaction | .05 | .03 | 1.87 | .062 | .05 | | .03 | 1.89 | .059 | **.03** | | **2.01** | **.044** |  | |  |  |  |

Note. All GIM tests were calculated with the robustSE package in R using 100 new datasets and 100 bootstraps, respectively. RoT = Rule of thumb test for necessity for GIM test (GIM test necessary if RoT>1.5). Significant results are printed in bold.

Table S3.

Comparison of main analysis (multilevel regression), Generalized linear models (GLM) with and without robust standard errors and Generalized Information Matrix Test (GIM) results for all models involving positive beliefs about oneself

| Minority type | Predictor | Multilevel model results | | | | GLM results | | | | GLM with robust SE | | | GIM | | |
| --- | --- | --- | --- | --- | --- | --- | --- | --- | --- | --- | --- | --- | --- | --- | --- |
|  |  | b | SE | T | p | b | SE | T | p | Robust SE | Z | p | RoT | GIM test | p |
| Sexual identity/orientation | Minority status | **5.95** | **.64** | **9.35** | **<.001** | **5.90** | **.65** | **9.06** | **<.001** | **1.42** | **4.12** | **<.001** | 5.00 | 2.4*10^-5^ | ≈ 1.00 |
|  | Pos. beliefs (self) | **-.18** | **.03** | **-5.48** | **<.001** | **-.22** | **.03** | **-7.04** | **<.001** | **.06** | **-3.79** | **<.001** |  |  |  |
|  | Interaction | **.22** | **.09** | **2.39** | **.017** | **.21** | **.09** | **2.29** | **.022** | .16 | 1.28 | .199 |  |  |  |
| Ethnicity | Minority status | **5.76** | **.67** | **8.58** | **<.001** | **5.91** | **.68** | **8.65** | **<.001** | **.98** | **6.01** | **<.001** | 4.75 | 1.9*10^-5^ | ≈ 1.00 |
|  | Pos. beliefs (sel ) | **-.19** | **.03** | **-5.90** | **<.001** | **-.23** | **.03** | **-7.38** | **<.001** | **.06** | **-4.12** | **<.001** |  |  |  |
|  | Interaction | **.27** | **.10** | **2.72** | **.007** | **.26** | **.10** | **2.54** | **.011** | **.13** | **1.99** | **.046** |  |  |  |
| Religion | Minority status | **5.23** | **.65** | **8.03** | **<.001** | **5.41** | **.66** | **8.14** | **<.001** | **1.13** | **4.76** | **<.001** | 4.37 | 1.8*10^-5^ | ≈ 1.00 |
|  | Pos. beliefs (self) | **-.17** | **.03** | **-5.19** | **<.001** | **-.21** | **.03** | **-6.80** | **<.001** | **.05** | **-4.41** | **<.001** |  |  |  |
|  | Interaction | .10 | .09 | 1.08 | .280 | .10 | .10 | 1.06 | .290 | .06 | 1.58 | .115 |  |  |  |
| Physical disability | Minority status | **2.62** | **.62** | **4.23** | **<.001** | **2.82** | **.63** | **4.48** | **<.001** | **1.21** | **2.33** | **.020** | 5.34 | 1.7*10^-5^ | ≈ 1.00 |
|  | Pos. beliefs (self) | **-.16** | **.03** | **-4.80** | **<.001** | **-.20** | **.03** | **-6.21** | **<.001** | **.06** | **-3.63** | **<.001** |  |  |  |
|  | Interaction | .08 | .09 | .87 | .386 | .04 | .09 | .48 | .633 | .05 | .92 | .359 |  |  |  |
| Visible physical difference | Minority status | **3.17** | **.52** | **6.06** | **<.001** | **3.06** | **.53** | **5.76** | **<.001** | **1.15** | **2.67** | **.007** | 4.86 | 2.1*10^-5^ | ≈ 1.00 |
|  | Pos. beliefs (self) | **-.13** | **.03** | **-3.96** | **<.001** | **-.19** | **.03** | **-5.70** | **<.001** | **.06** | **-3.12** | **.002** |  |  |  |
|  | Interaction | .00 | .08 | .00 | .999 | .02 | .08 | .20 | .844 | .15 | .103 | .918 |  |  |  |
| Intersectionality index | Intersect. index | **2.67** | **.21** | **12.78** | **<.001** | **2.71** | **.21** | **12.73** | **<.001** | **.14** | **19.38** | **<.001** | 4.36 | 1.9*10^-5^ | ≈ 1.00 |
|  | Pos. beliefs (self) | **-.17** | **.04** | **-4.73** | **<.001** | **-.22** | **.04** | **-6.14** | **<.001** | **.06** | **-3.75** | **<.001** |  |  |  |
|  | Interaction | **.07** | **.03** | **2.44** | **.015** | **.07** | **.03** | **2.29** | **.022** | **.03** | **2.93** | **.042** |  |  |  |

Note. All GIM tests were calculated with the robustSE package in R using 100 new datasets and 100 bootstraps, respectively. RoT = Rule of thumb test for necessity for GIM test (GIM test necessary if RoT>1.5). Significant results are printed in bold.

Table S4.

Comparison of main analysis (multilevel regression), Generalized linear models (GLM) with and without robust standard errors and Generalized Information Matrix Test (GIM) results for all models involving positive beliefs about others

| Minority type | Predictor | Multilevel model results | | | | GLM results | | | | GLM with robust SE | | | GIM | | |
| --- | --- | --- | --- | --- | --- | --- | --- | --- | --- | --- | --- | --- | --- | --- | --- |
|  |  | b | SE | T | p | b | SE | T | p | Robust SE | Z | p | RoT | GIM test | p |
| Sexual identity/orientation | Minority status | **5.87** | **.64** | **9.22** | **<.001** | **5.82** | **.65** | **8.94** | **<.001** | **1.53** | **3.81** | **<.001** | 5.07 | 2.1*10^-5^ | ≈ 1.00 |
|  | Pos. beliefs (other) | **-.29** | **.03** | **-8.93** | **<.001** | **-.33** | **.03** | **-9.87** | **<.001** | **.04** | **-9.29** | **<.001** |  |  |  |
|  | Interaction | **.28** | **.10** | **2.98** | **.003** | **.29** | **.09** | **2.94** | **.003** | **.13** | **2.05** | **.041** |  |  |  |
| Ethnicity | Minority status | **5.60** | **.67** | **8.31** | **<.001** | **5.78** | **.69** | **8.43** | **<.001** | **1.10** | **5.27** | **<.001** | 4.77 | 1.2*10^-5^ | ≈ 1.00 |
|  | Pos. beliefs (other) | **-.29** | **.03** | **-8.73** | **<.001** | **-.32** | **.03** | **-9.75** | **<.001** | **.03** | **-9.79** | **<.001** |  |  |  |
|  | Interaction | .20 | .10 | 1.92 | .054 | **.23** | **.11** | **2.23** | **.026** | .17 | 1.36 | .172 |  |  |  |
| Religion | Minority status | **5.05** | **.67** | **7.57** | **<.001** | **5.19** | **.68** | **7.63** | **<.001** | **1.23** | **4.21** | **<.001** | 4.46 | 1.9*10^-5^ | ≈ 1.00 |
|  | Pos. beliefs (other) | **-.28** | **.03** | **-8.40** | **<.001** | **-.31** | **.03** | **-9.37** | **<.001** | **.04** | **-7.88** | **<.001** |  |  |  |
|  | Interaction | .19 | .10 | 1.91 | .056 | .20 | .10 | 1.95 | .052 | .15 | 1.33 | .180 |  |  |  |
| Physical disability | Minority status | **2.50** | **.60** | **4.14** | **<.001** | **2.80** | **.61** | **4.56** | **<.001** | **1.31** | **2.14** | **.033** | 5.48 | 2.0*10^-5^ | ≈ 1.00 |
|  | Pos. beliefs (other) | **-.27** | **.03** | **-7.94** | **<.001** | **-.30** | **.03** | **-8.78** | **<.001** | **.05** | **-5.61** | **<.001** |  |  |  |
|  | Interaction | -.03 | .09 | -.29 | .772 | -.04 | .09 | -.44 | .660 | .07 | -.55 | .579 |  |  |  |
| Visible physical difference | Minority status | **3.00** | **.51** | **5.84** | **<.001** | **2.88** | **.52** | **5.51** | **<.001** | **1.09** | **2.64** | **.008** | 5.09 | 2.0*10^-5^ | ≈ 1.00 |
|  | Pos. beliefs (other) | **-.25** | **.03** | **-7.27** | **<.001** | **-.29** | **.04** | **-8.19** | **<.001** | **.07** | **-4.41** | **<.001** |  |  |  |
|  | Interaction | -.04 | .08 | -.55 | .584 | .04 | .08 | -.53 | .594 | .17 | -.25 | .800 |  |  |  |
| Intersectionality index | Intersect. index | **2.58** | **.21** | **12.27** | **<.001** | **2.63** | **.21** | **12.26** | **<.001** | **.23** | **11-37** | **<.001** | 4.54 | 1.9*10^-5^ | ≈ 1.00 |
|  | Pos. beliefs (other) | **-.27** | **.04** | **-7.39** | **<.001** | **-.31** | **.04** | **-8.26** | **<.001** | **.04** | **-7.09** | **<.001** |  |  |  |
|  | Interaction | **.06** | **.03** | **2.03** | **.043** | **.07** | **.03** | **2.07** | **.038** | .04 | 1.81 | .070 |  |  |  |

Note. All GIM tests were calculated with the robustSE package in R using 100 new datasets and 100 bootstraps, respectively. RoT = Rule of thumb test for necessity for GIM test (GIM test necessary if RoT>1.5). Significant results are printed in bold.

Table S5

Comparison of main analysis (multilevel regression), Generalized linear models (GLM) with and without robust standard errors and Generalized Information Matrix Test (GIM) results for all models involving social rank

| Minority type | Predictor | Multilevel model results | | | | GLM results | | | | GLM with robust SE | | | GIM | | |
| --- | --- | --- | --- | --- | --- | --- | --- | --- | --- | --- | --- | --- | --- | --- | --- |
|  |  | b | SE | T | p | b | SE | T | p | Robust SE | Z | p | RoT | GIM test | p |
| Sexual identity/orientation | Minority status | **5.99** | **.63** | **9.44** | **<.001** | **5.93** | **.64** | **9.15** | **<.001** | **1.47** | **4.04** | **<.001** | 4.92 | 2.3*10^-5^ | ≈ 1.00 |
|  | Social rank | **-.07** | **.01** | **-7.00** | **<.001** | **-.08** | **.01** | **-8.50** | **<.001** | **.03** | **-2.66** | **.008** |  |  |  |
|  | Interaction | **.10** | **.03** | **3.51** | **<.001** | **.10** | **.03** | **3.43** | **<.001** | .06 | 1.60 | .110 |  |  |  |
| Ethnicity | Minority status | **5.75** | **.67** | **8.55** | **<.001** | **5.87** | **.68** | **8.58** | **<.001** | **1.07** | **5.50** | **<.001** | 4.68 | 2.2*10^-5^ | ≈ 1.00 |
|  | Social rank | **-.07** | **.01** | **-6.70** | **<.001** | **-.08** | **.01** | **-8.15** | **<.001** | **.03** | **-2.67** | **<.007** |  |  |  |
|  | Interaction | **.07** | **.03** | **2.15** | **.031** | **.07** | **.03** | **2.17** | **.030** | .06 | 1.08 | .282 |  |  |  |
| Religion | Minority status | **5.12** | **.65** | **7.83** | **<.001** | **5.26** | **.67** | **7.89** | **<.001** | **1.11** | **.76** | **.001** | 4.30 | 3.6*10^-5^ | ≈ 1.00 |
|  | Social rank | **-.06** | **.01** | **-6.00** | **<.001** | **-.08** | **.01** | **-7.52** | **<.001** | **.03** | **-2.64** | **.008** |  |  |  |
|  | Interaction | .02 | .03 | .68 | .495 | .02 | .03 | .66 | .510 | .03 | .62 | .540 |  |  |  |
| Physical disability | Minority status | **2.36** | **.62** | **3.78** | **<.001** | **2.49** | **.63** | **3.93** | **<.001** | 1.31 | 1.90 | .057 | 5.33 | 2.3*10^-5^ | ≈ 1.00 |
|  | Social rank | **-.06** | **.01** | **-5.44** | **<.001** | **-.07** | **.01** | **-6.64** | **<.001** | **.03** | **-2.64** | **.008** |  |  |  |
|  | Interaction | -.01 | .03 | -.25 | .800 | -.02 | .03 | .81 | .419 | .02 | -1.00 | .317 |  |  |  |
| Visible physical difference | Minority status | **3.07** | **.52** | **5.90** | **<.001** | **2.95** | **.53** | **5.58** | **<.001** | **1.05** | **2.82** | **.004** | 4.78 | 1.8*10^-5^ | ≈ 1.00 |
|  | Social rank | **-.05** | **.01** | **-4.95** | **<.001** | **-.07** | **.01** | **-6.45** | **<.001** | **.03** | **-2.07** | **.039** |  |  |  |
|  | Interaction | -.01 | .02 | -.36 | .722 | -.01 | .03 | -049 | .622 | .04 | -.30 | .760 |  |  |  |
| Intersectionality index | Intersect. index | **2.60** | **.21** | **12.44** | **<.001** | **2.63** | **.21** | **12.34** | **<.001** | **.21** | **12.69** | **<.001** | 4.30 | 3.7*10^-5^ | ≈ 1.00 |
|  | Social rank | **-.06** | **.01** | **-4.96** | **<.001** | **-.07** | **.01** | **-6.16** | **<.001** | **.03** | **-2.08** | **.038** |  |  |  |
|  | Interaction | .01 | .01 | 1.43 | .153 | .01 | .01 | 1.14 | .255 | .02 | .67 | .504 |  |  |  |

Note. All GIM tests were calculated with the robustSE package in R using 100 new datasets and 100 bootstraps, respectively. RoT = Rule of thumb test for necessity for GIM test (GIM test necessary if RoT>1.5). Significant results are printed in bold.
